# ATP-binding cassette protein ABCA7 deficiency impairs sphingomyelin synthesis, cognitive discrimination, and synaptic plasticity in the entorhinal cortex

**DOI:** 10.1016/j.jbc.2022.102411

**Published:** 2022-08-23

**Authors:** Jahangir Iqbal, Manuel D. Suarez, Pradeep K. Yadav, Meghan T. Walsh, Yimeng Li, Yiyang Wu, Zhengwei Huang, Antonisamy William James, Victor Escobar, Ashwag Mokbe, Adam M. Brickman, José A. Luchsinger, Kezhi Dai, Herman Moreno, M. Mahmood Hussain

**Affiliations:** 1 Department of Cell Biology, SUNY Downstate Medical Center, Brooklyn, New York, USA; 2 King Abdullah International Medical Research Center, King Saud bin Abdulaziz University for Health Sciences, Ministry of National Guard Health Affairs, Al Ahsa, Saudi Arabia; 3 Departments of Neurology and Physiology/Pharmacology, The Robert F. Furchgott Center for Neural and Behavioral Science, SUNY Downstate Medical Center, and Kings County Hospital, Brooklyn, New York, USA; 4 Department of Foundations of Medicine, NYU Long Island School of Medicine, Mineola, New York, USA; 5 Institute of Mental Health, The Affiliated Kangning Hospital of Wenzhou Medical University, Wenzhou, China; 6 Taub Institute for Research on Alzheimer’s Disease and the Aging Brain and Department of Neurology, College of Physicians and Surgeons, Columbia University, New York, New York, USA; 7 Departments of Medicine and Epidemiology, Columbia University Irving Medical Center, New York, New York, USA; 8 School of Mental Health, Wenzhou Medical University, Wenzhou, China

**Keywords:** Alzheimer Disease, sphingomyelin, ABCA7, sphingolipids, lipids, APA, active place avoidance, BSA, bovine serum albumin, FBS, fetal bovine serum, fEPSP, field excitatory postsynaptic potentials, HDL, high-density lipoprotein, HFS, high frequency stimulation, KD, knockdown, LEC, lateral entorhinal cortex, LOAD, late-onset Alzheimer’s Disease, LTP, long-term potentiation, MTP, microsomal triglyceride transfer protein, PPR, paired-pulse ratio, SM, sphingomyelin

## Abstract

Sphingomyelin (SM) is an abundant plasma membrane and plasma lipoprotein sphingolipid. We previously reported that ATP-binding cassette family A protein 1 (ABCA1) deficiency in humans and mice decreases plasma SM levels. However, overexpression, induction, downregulation, inhibition, and knockdown of ABCA1 in human hepatoma Huh7 cells did not decrease SM efflux. Using unbiased siRNA screening, here, we identified that ABCA7 plays a role in the biosynthesis and efflux of SM without affecting cellular uptake and metabolism. Since loss of function mutations in the *ABCA7* gene exhibit strong associations with late-onset Alzheimer’s disease across racial groups, we also studied the effects of ABCA7 deficiency in the mouse brain. Brains of *ABCA7*-deficient (KO) mice, compared with WT, had significantly lower levels of several SM species with long chain fatty acids. In addition, we observed that older KO mice exhibited behavioral deficits in cognitive discrimination in the active place avoidance task. Next, we performed synaptic transmission studies in brain slices obtained from older mice. We found anomalies in synaptic plasticity at the intracortical synapse in layer II/III of the lateral entorhinal cortex but not in the hippocampal CA3-CA1 synapses in KO mice. These synaptic abnormalities in KO brain slices were rescued with extracellular SM supplementation but not by supplementation with phosphatidylcholine. Taken together, these studies identify a role of ABCA7 in brain SM metabolism and the importance of SM in synaptic plasticity and cognition, as well as provide a possible explanation for the association between ABCA7 and late-onset Alzheimer’s disease.

Sphingolipids are biologically active lipids involved in several biological processes (1, 2). Sphingomyelin (SM), a major sphingolipid, is abundant in the outer leaflet of cell membranes where it forms lipid rafts with cholesterol. Additionally, SM is the most abundant sphingolipid in plasma lipoproteins (3). We previously showed that microsomal triglyceride transfer protein (MTP) and ATP-binding cassette transporter family A protein 1 (ABCA1) play important roles in determining plasma concentrations of SM (4, 5). Mechanistic studies showed that both of these proteins do not affect SM synthesis. It is known that MTP transfers lipids *in vitro* between vesicles and possibly to apoB *in vivo* to assist in lipoprotein assembly and secretion (6, 7). We showed that MTP transfers SM *in vitro* and suggested that it might also do so in cells and facilitate secretion of SM with lipoproteins to explain significant reductions in plasma SM levels in MTP-deficient humans and mice (4). ABCA1 is involved in the efflux of phospholipids and cholesterol to high-density lipoproteins (HDLs) and its deficiency in humans and mice is associated with low levels of plasma HDL. Although chronic deficiency of ABCA1 significantly reduced plasma SM levels in humans and mice, acute knockdown or inhibition had no effect on SM efflux from human hepatoma cells (5). Therefore, we surmised that ABCA1 plays a role in the biogenesis of HDL, and HDL is involved in the efflux of SM from cells involving an unexplained mechanism. Here, using an unbiased siRNA-mediated knockdown (KD) approach, we identified that ABCA7 plays a role in the biosynthesis and efflux of SM. Since *ABCA7* gene has been associated with late-onset Alzheimer’s disease (LOAD) (8), we addressed the role of ABCA7 in the brain. We observed that ABCA7 knockout (*Abca7*^*−/−*^) mice have reduced levels of certain species of SM in the brain and that ABCA7 deficiency affects cognitive discrimination and synaptic plasticity in brain slices which can be mitigated by supplementation of SM.

## Results

### ABCA7 participates in SM efflux

**Figure 1:**
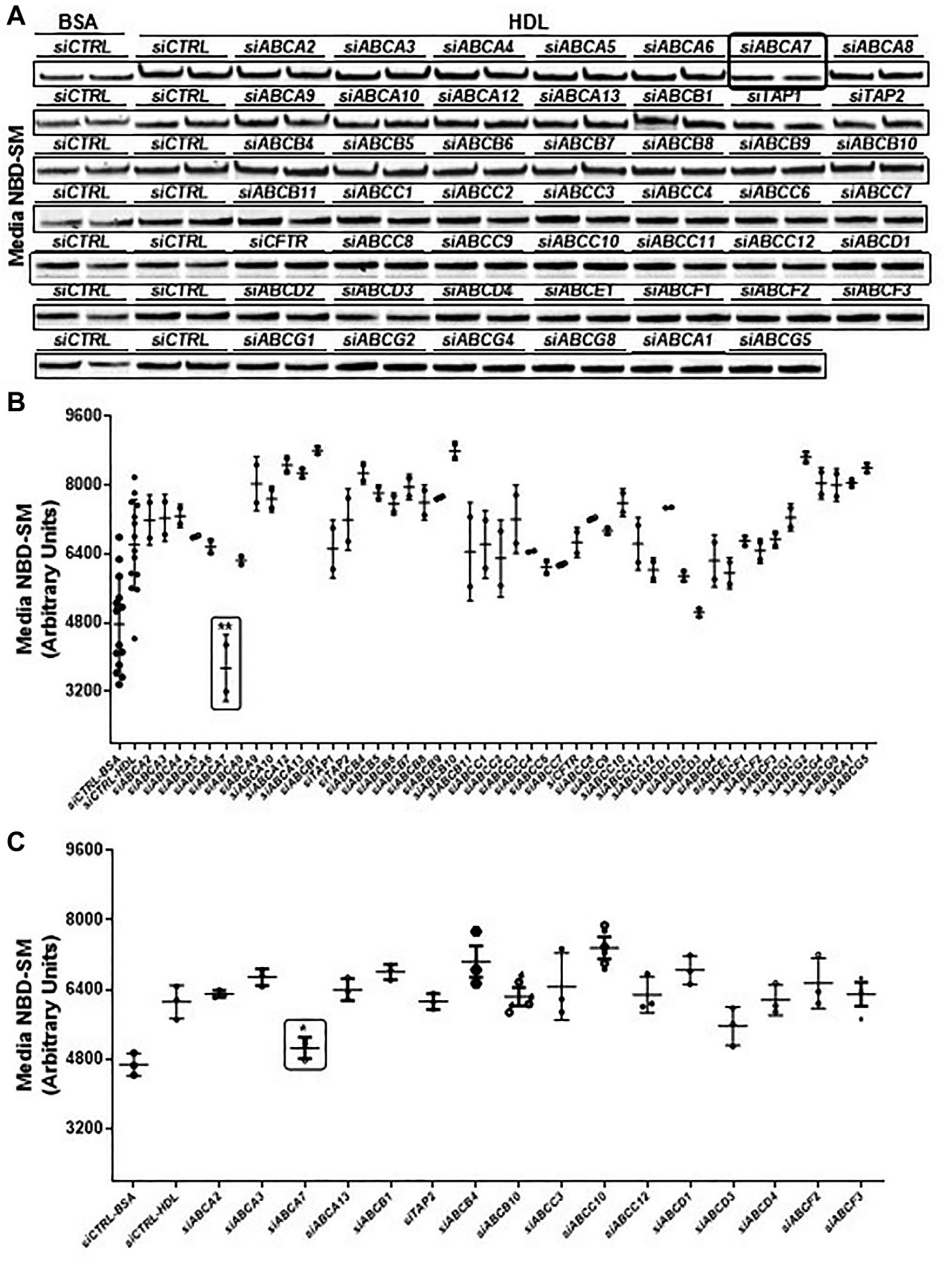
**Primary and secondary screening to identify membrane transporter involved in SM efflux to HDL.***A* and *B*, primary screening to identify transporter that decrease SM efflux. Huh7 cells were transfected in duplicate with 50 nM of siCTRL or siRNA against various indicated membrane transporters for 72 h, incubated with 2 μM of C6-NBD Cer in DMEM containing 10% FBS for 3 h, washed three times with DMEM containing 0.1% BSA, and then incubated with either 40 μg/ml BSA or HDL in DMEM for 6 h to study SM efflux. Lipids in the media were extracted and separated on TLC, and fluorescence in SM bands was visualized with a PhosphorImager (*A*), quantified in ImageJ software and plotted (*B*). *C*, secondary screening to identify SM transporter. A second screening of selected transporters from the first screening was performed as described above, in triplicate. Values are plotted as mean ± SD, ∗*p* < 0.05 and ∗∗*p* < 0.01 by *t* test, as compared with *siCTRL-HDL*–treated cells. BSA, bovine serum albumin; FBS, fetal bovine serum; HDL, high-density lipoprotein; SM, sphingomyelin.

We previously reported that ATP-binding cassette family A protein 1 (ABCA1) deficiency in humans and mice decreases plasma SM levels (5). However, overexpression, induction, downregulation, inhibition, and KD of ABCA1, a well-characterized cholesterol and phospholipid transporter, in human hepatoma Huh7 cells does not decrease SM efflux to HDL (5). Therefore, we hypothesized that ABCA1 is not directly involved in the SM efflux to HDL but contributes to plasma SM levels by playing a role in the biogenesis of HDL. To identify the SM transporter, we individually knocked down 48 members of the ABC transporter family by using an siRNA library in Huh7 cells, which were treated with C6-NBD ceramide (Cer). Ceramide is taken up by cells and is converted to sphingomyelin (SM) and glucosylceramide (GluCer). After labeling, cells were incubated with bovine serum albumin (BSA) to quantify basal efflux (siCTRL-BSA) or efflux to HDL (siCTRL-HDL) (Fig. 1). Compared with BSA, HDL significantly increased SM efflux (Fig. 1, *A* and *B*), in agreement with findings from our earlier studies (5), thus confirming that HDL enhances SM efflux. KD of different genes either increased or decreased SM efflux to HDL (Fig. 1, *A* and *B*). Of interest was one protein, ABCA7, whose KD significantly decreased SM efflux to HDL (Fig. 1, *A* and *B*). We validated these results by performing a second screening. In agreement with the results of our first screening, incubation of siCTRL-treated cells with HDL resulted in significantly greater SM efflux than that with siCTRL-BSA, and this enhanced efflux significantly decreased after KD of ABCA7 (Fig. 1*C*). These results suggested that ABCA7 might be involved in the efflux of SM to HDL.

### ABCA7 KD in Huh7 cells decreases SM synthesis

**Figure 2:**
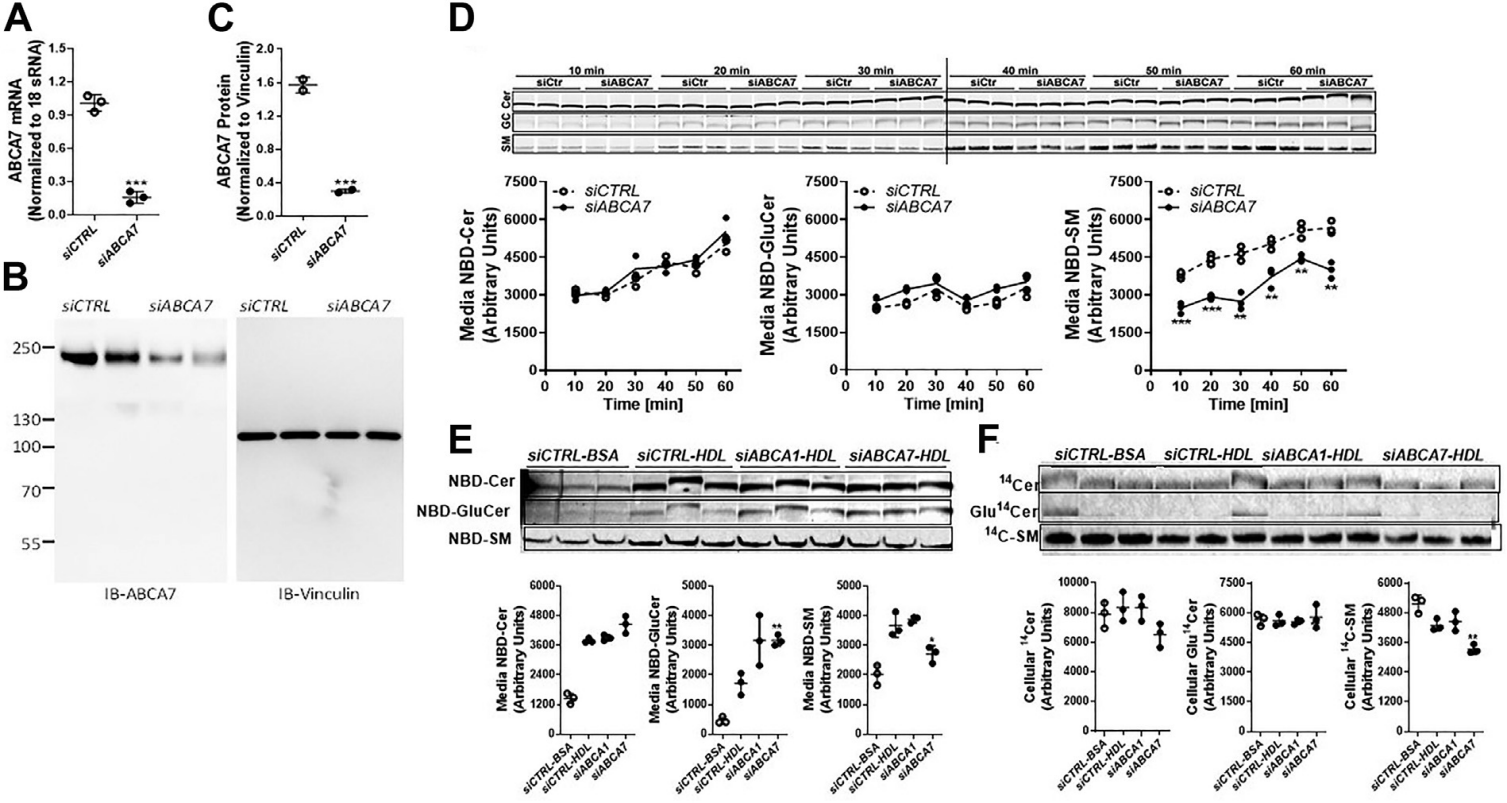
**Synthesis of SM is decreased after ABCA7 KD.***A*–*C*, knockdown of ABCA7 using siRNAs. Huh-7 cells were transfected in triplicate wells with siCTRL or siRNA and amounts of ABCA7 mRNA were quantified (*A*). In addition, cell lysates were separated on gel and immunoblotted (IB-ABCA7) with ABCA7 antibodies and anti-vinculin (anti-vinculin) (*B*). Positions of PageRuler Plus Stained protein ladder (Invitrogen, #26619) molecular weight markers are shown in the *left*. Vinculin was used as a control. Blots were quantified by image J and normalized values were plotted (*C*). Values are mean + SD, *t* test, ∗∗∗*p* < 0.001. *D*, reduced SM synthesis in *siABCA7*-treated cells. Huh7 cells were transfected in triplicate with 50 nM of *siCTRL* or *siABCA7* for 72 h, labeled with 2 μM of C6-NBD Cer in 10% FBS for different time points, and washed three times with 0.1% BSA in DMEM. Lipids in cells were extracted at different times and separated on TLC. Fluorescence in Cer, GluCer, and SM bands was visualized with a PhosphorImager (*top*) and quantified using ImageJ software (*bottom*). Values are mean ± SD, *t* test, ∗∗*p* < 0.01 and ∗∗∗*p* < 0.001 compared with *siCTRL*-treated cells. *E* and *F*, ABCA7 KD decreases synthesis and efflux of [^14^C]-SM to HDL. Huh7 cells were transfected in triplicate with 50 nM of *siCTRL*, *siABCA1*, or *siABCA7* for 72 h. Cells were labeled with 0.2 μCi of [^14^C]-Cer in DMEM containing 10% FBS for 3 h, washed three times with DMEM containing 0.1% BSA, and incubated with either 40 μg/ml BSA or HDL for 6 h. Lipids in the (*E*) media and (*F*) cells were extracted and separated on TLC. Radioactive Cer, GluCer, and SM bands were visualized with a PhosphorImager (*top*) and quantified in ImageJ software (*bottom*). Values are plotted as replicates (mean ± SD). ∗*p* < 0.05 and ∗∗*p* < 0.01 compared with *siCtrl-HDL*–treated cells. BSA, bovine serum albumin; FBS, fetal bovine serum; HDL, high-density lipoprotein; SM, sphingomyelin.

In previous studies, we showed that MTP and ABCA1 deficiencies have no effect on SM synthesis; however, they were involved in secretion and efflux, respectively (4, 5). Therefore, we hypothesized that ABCA7 would not affect SM synthesis but would decrease efflux. To test this hypothesis, we transfected Huh7 cells with *siABCA7* to knockdown (KD) ABCA7 by more than 80% compared to siCTRL-treated cells (Fig. 2, *A*–*C*). These cells were then incubated with C6-NBD Cer for 10 to 60 min. Measurement of intracellular sphingolipids revealed that cellular Cer levels increased with time, thus indicating time-dependent uptake (Fig. 2*D*). At the earliest time point studied, NBD-Cer had already been converted to GluCer, and the levels remained similar over 1 h in both *siCTRL*- and *siABCA7*-treated cells. In contrast, conversion of Cer to SM increased with time in both *siCTRL*- and *siABCA7*-treated cells. However, unexpectedly, these time-dependent increases were significantly lower in ABCA7 KD cells, thereby leading to lower intracellular SM levels than those in *siCTRL*-treated cells (Fig. 2*D*). These studies suggest that ABCA7 is involved in SM synthesis but not in the synthesis of GluCer.

We considered the possibility that decreased synthesis of SM might have been an artifact due to the use of NBD-labeled C6-Cer as a precursor for SM synthesis. Therefore, we labeled *siCTRL*- or *siABCA7*-treated Huh7 cells with C16-[^14^C]-Cer and measured labeled sphingolipids in the media and cells. For an additional control, we performed KD of ABCA1, whose deficiency decreases plasma SM levels in mice and humans (5). First, we quantified the efflux of [^14^C]-sphingolipids to BSA and HDL in the media (Fig. 2*E*). We observed significantly greater levels of Cer, GluCer, and SM in the media of *siCTRL-HDL*– than *siCTRL-BSA*–treated cells. *SiABCA1* had no significant effect on the efflux of Cer, GluCer, and SM to HDL. We did not observe any difference in the efflux of Cer to HDL between *siABCA7*-treated cells and *siCTRL-HDL*–treated cells. However, we observed significantly greater efflux of GluCer and significantly less efflux of SM to HDL in *siABCA7-HDL* than in *siCTRL-HDL*–treated cells. These studies indicated that *siABCA7* significantly decreases [^14^C]-SM efflux to HDL. Next, we measured sphingolipids in cells (Fig. 2*F*). *SiABCA1* and *siABCA7*, compared with *siCTRL-HDL*, had no significant effect on cellular Cer and GluCer levels. In contrast, *siABCA7*-treated cells had significantly lower levels of radiolabeled SM than *siCTRL*- and *siABCA1*-treated cells, thus indicating decreased synthesis.

To determine whether ABCA7 might play a role in both synthesis and efflux or whether decreased efflux might be secondary to lower synthesis, we calculated the efflux as a percentage of the total SM level present in the cells and media (Table 1). *SiABCA1* had no effect on Cer and SM efflux but increased GluCer efflux. In contrast, *siABCA7* increased the efflux of Cer and GluCer but had no effect on SM efflux. Based on these calculations, we surmised that ABCA7 plays a role in SM synthesis, and decreased efflux is secondary to lower synthesis and lower cellular SM levels. Because of reduced SM synthesis in the absence of ABCA7, more Cer is likely to remain in cells, and conversion to GluCer is likely to be greater; therefore, these lipids should be available for efflux to HDL. Together, these studies indicate that KD of ABCA7 significantly decreases the conversion of [^14^C]-Cer to SM and the efflux of [^14^C]-SM to HDL.

**Table 1:** Efflux of different sphingolipids to HDL as a percentage of total lipids

	Cellular (AU)	Media (AU)	Total (AU)	% Efflux	*p* value
[^14^C]-Ceramide					
Ceramide					
siCTRL-BSA	7861 ± 868	1447 ± 214	9307 ± 788	15.7 ± 2.9	
siCTRL-HDL	8343 ± 993	3791 ± 98	12,134 ± 978	31.4 ± 2.7	
siABCA1-HDL	8312 ± 842	3904 ± 123	12,216 ± 902	32.1 ± 2.1	NS
siABCA7-HDL	6504 ± 807	4447 ± 355	10,951 ± 737	40.7 ± 4.2	<0.05
Glucosylceramide					
siCTRL-BSA	5695 ± 297	464 ± 110	6123 ± 347	7.6 ± 1.5	
siCTRL-HDL	5601 ± 288	1711 ± 369	7312 ± 599	23.2 ± 3.4	
siABCA1-HDL	5553 ± 131	3166 ± 851	8719 ± 978	35.9 ± 5.8	<0.05
siABCA7-HDL	5779 ± 600	3165 ± 184	8945 ± 784	35.4 ± 1.0	<0.005
Sphingomyelin					
siCTRL-BSA	5165 ± 347	2008 ± 333	7173 ± 676	27.9 ± 2.1	
siCTRL-HDL	4300 ± 243	3674 ± 409	7974 ± 354	46.0 ± 3.6	
siABCA1-HDL	4455 ± 394	3878 ± 110	8332 ± 486	46.6 ± 1.7	NS
siABCA7-HDL	3326 ± 184	2702 ± 281	6028 ± 134	44.8 ± 3.9	NS

Data from Figure 2, *B* and *C* were used to calculate the percentage efflux with respect to total sphingolipid arbitrary units present in the media and cells at the end of the experiment. Statistical differences were calculated in comparison to siCTRL-HDL *via* Student’s *t* test.

Abbreviations: AU, arbitrary units; NS, not significant.

### ABCA7 deficiency does not affect SM uptake and metabolism

The above studies showed that ABCA7 is involved in SM synthesis. We also considered several other possible roles of ABCA7 in SM synthesis. It has been shown that ceramide transfer protein 1 (CERT1) is important for SM synthesis (9). Therefore, we asked whether CERT1 expression is altered in *Abca7* deficiency and measured ABCA7 and CERT1 mRNA levels in the livers of WT and *Abca7*^*−/−*^-deficient (KO) mice. Compared to WT, KO livers had barely detectable ABCA7 mRNA levels confirming ABCA7 deficiency (Fig. S1). In contrast, ABCA7 deficiency had no effect on CERT1 mRNA levels. These studies showed that ABCA7 deficiency has no effect on CERT1 expression.

**Figure 3:**
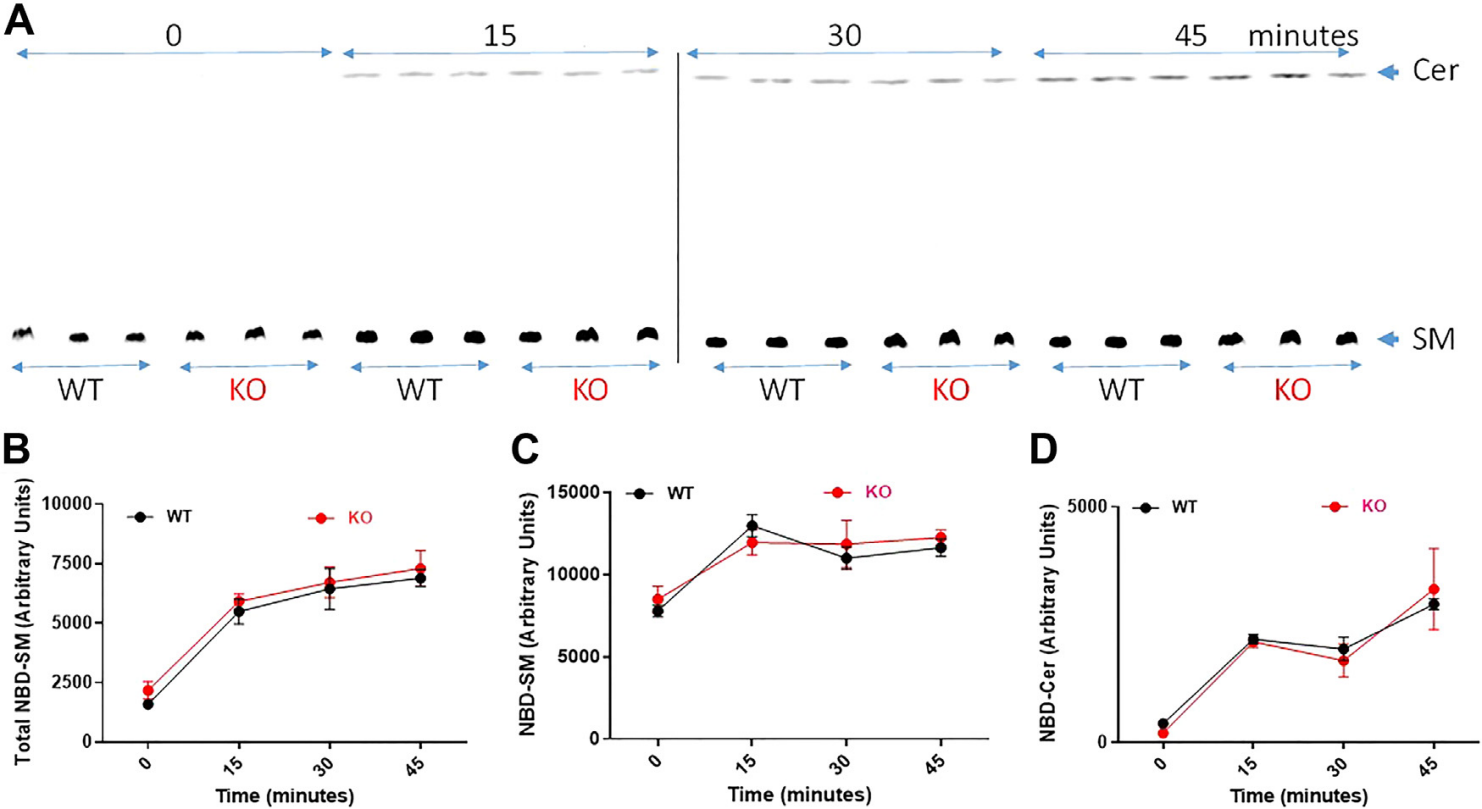
**Time-dependent uptake of NBD-SM by WT and KO primary hepatocytes.** Primary hepatocytes from WT and ABCA7 KO mice were isolated and cultured in triplicate wells. After 24 h, cells were incubated with 2 μM C6-NBD SM in HepotoZYME-SFM containing 10% FBS for different indicated time points and washed three times with PBS before extracting lipids using isopropanol. The extracted lipids were divided into two portions. One portion was used for TLC (*A*) and other portion was used for direct fluorescence measurements (*B*). Fluorescent NBD-SM and NBD-Cer bands on TLC plates were visualized using a PhosphorImager (*A*) and quantified using Image J software (*C* and *D*). Significance was determined by two-way ANOVA and error bars represent mean ± SD. No significant differences were observed in the uptake of NBD-SM. FBS, fetal bovine serum; SM, sphingomyelin.

Next, we studied the uptake of NBD-labeled SM by these cells (Fig. 3, *A*–*D*). Both WT and KO hepatocytes took up similar amounts of NBD-SM fluorescence (Fig. 3*B*). Moreover, both cells showed similar uptake of NBD-SM (Fig. 3, *A* and *C*). We observed that there was an increase in NBD-Cer in cells with time (Fig. 3*A*). The production of ceramide was similar in WT and KO hepatocytes (Fig. 3, *A* and *D*). These studies showed that hepatocytes take up NBD-SM and a small portion is hydrolyzed to ceramide. Both uptake of SM and conversion to ceramide were unaffected by ABCA7 deficiency.

**Figure 4:**
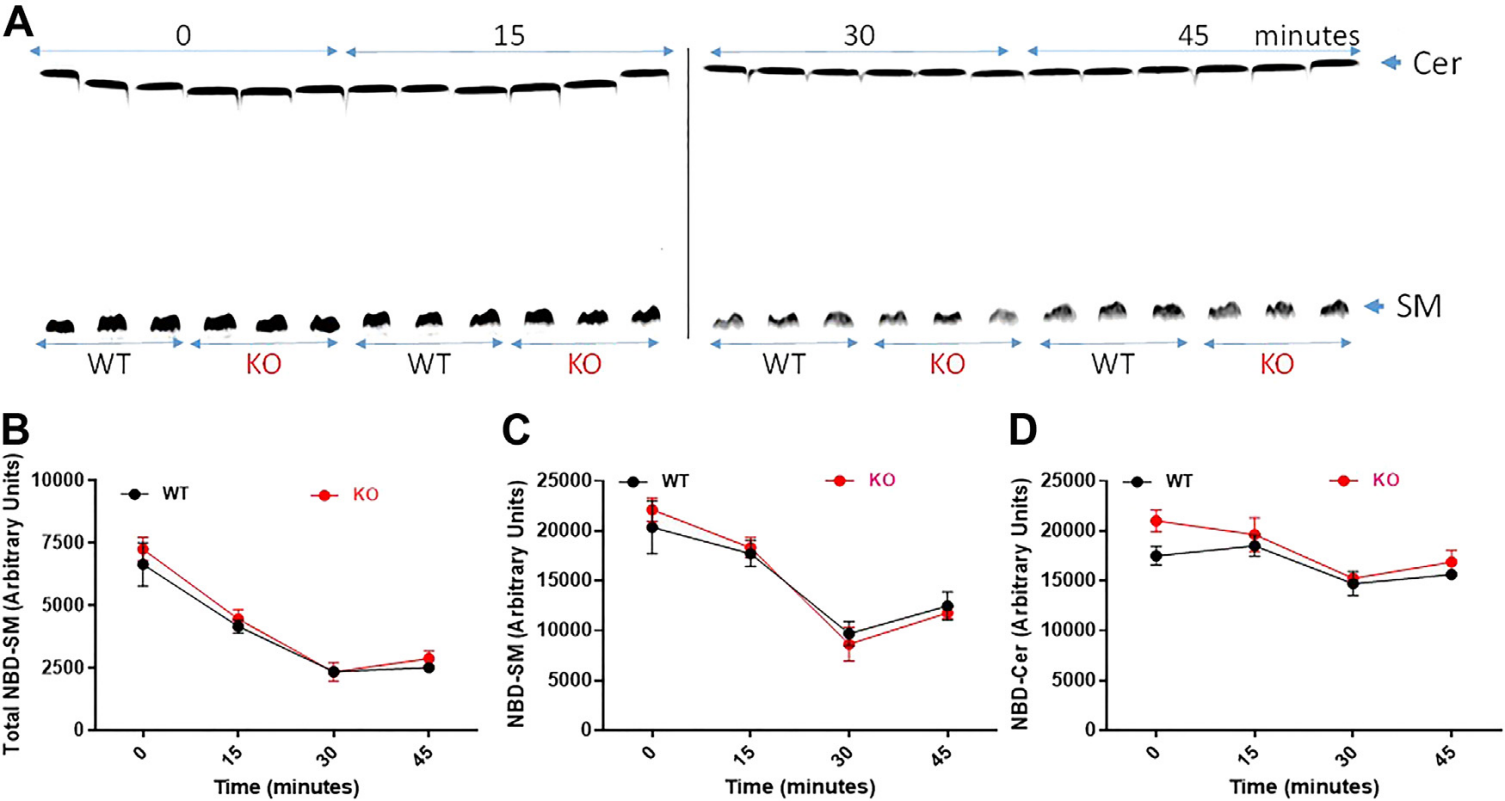
**Sphingomyelin metabolism in primary hepatocytes.** Primary hepatocytes from WT and ABCA7 KO mice were incubated with 2 μM C6-NBD SM for 4 h in triplicates. Cells were washed and supplemented with media without C6-NBD SM. At indicated times, lipids were extracted and divided into two portions and used for TLC separation (*A*) and direct quantification of fluorescence (*B*). SM and Cer bands on the TLC plates were visualized using a PhosphorImager (*A*). Furthermore, SM (*C*) and ceramide (*D*) bands were quantified using Image J. Significance was determined with two-way ANOVA and error bars represent mean ± SD. No significant differences were found in the degradation of NBD-SM in WT and KO hepatocytes. SM, sphingomyelin.

To investigate further the role of ABCA7 in the hydrolysis of SM, we first labeled WT and KO hepatocytes with NBD-SM. Next, we studied time-dependent conversion of NBD-SM to NBD-Cer (Fig. 4). Total disappearance of NBD-SM was similar in both hepatocytes (Fig. 4*B*). We observed similar decline in cellular NBD-SM with time (Fig. 4, *A* and *C*). Moreover, the production of NBD-Cer was similar in WT and KO hepatocytes (Fig. 4, *A* and *D*). We did not see rapid increase in NBD-Cer levels corresponding to loss of cellular NBD-SM. These studies suggested that NBD-SM is metabolized to other unknown metabolites. Nevertheless, these studies showed that degradation of NBD-SM in hepatocytes was unaffected by the absence of ABCA7.

In short, these studies showed that mouse hepatocyte take up SM. A portion of it is converted to NBD-Cer probably due to sphingomyelinase activity. More importantly, we did not find any differences between WT and KO hepatocytes with respect to SM uptake and metabolism. Therefore, it is likely that ABCA7 does not play a major role in SM uptake and hydrolysis.

### An ABCA7 missense mutation found in patients with LOAD decreases SM synthesis and efflux in cells

**Figure 5:**
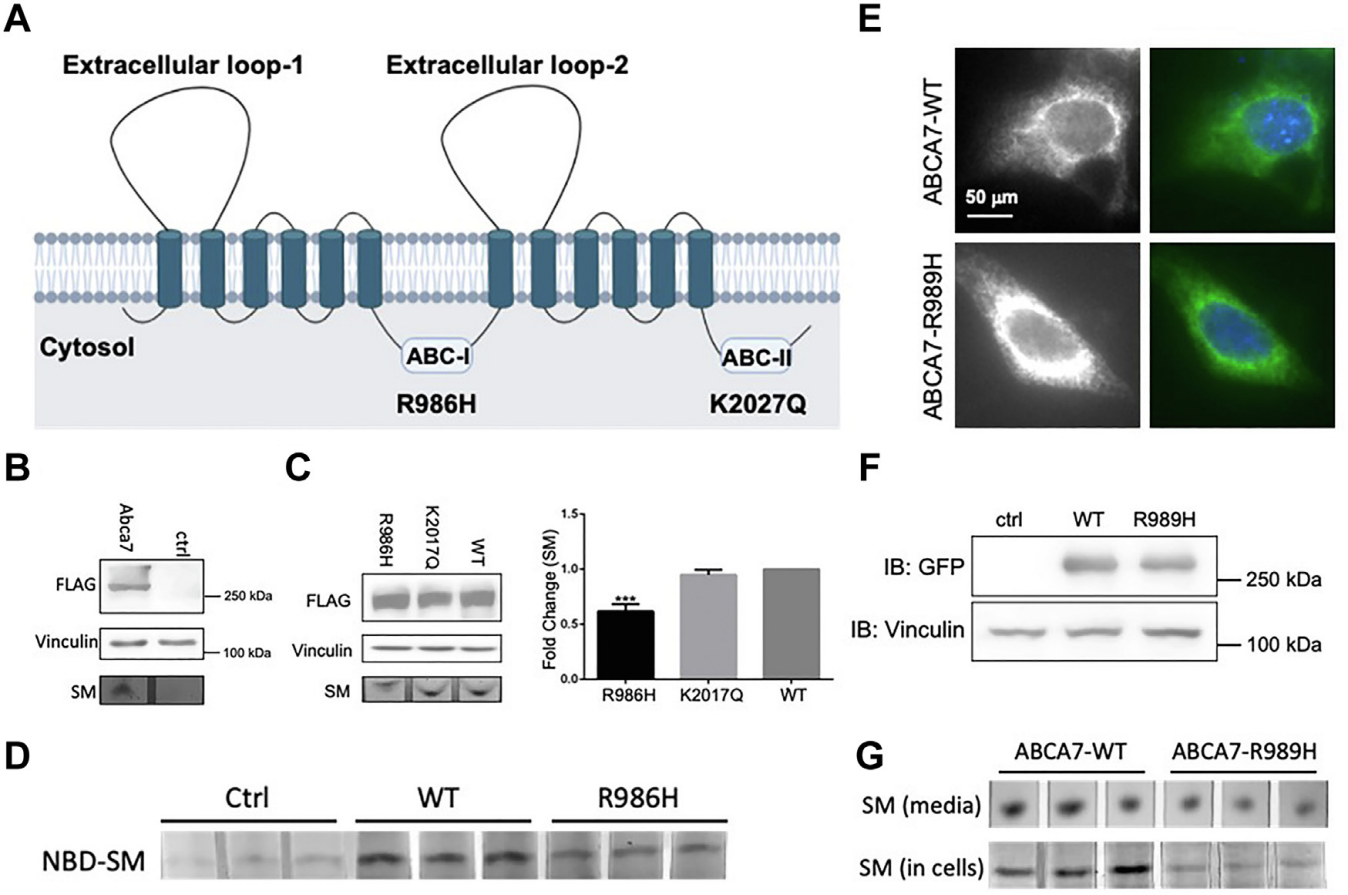
**Importance of the nucleotide-binding domain of ABCA7 in SM synthesis and efflux.***A*, schematic diagram depicting different structural domains of mouse ABCA7. R986H and K2027Q are in the ABC-I or ABC-II domains, respectively. *B*–*D*, effect of expression of WT and different missense mouse ABCA7 mutants on SM efflux in 293T cells. *B*, overexpression of ABCA7 in 293T cells increases SM efflux. Mouse WT ABCA7 protein was expressed as FLAG-tagged protein, and expression was determined with anti-FLAG antibodies (*top*). Vinculin was used as a loading control (*middle*). Cells were labeled with NBD-Cer for 4 h, washed, and incubated with DMEM containing 1% BSA for 2 h and then washed and incubated with 40 μg/ml of human HDL for 3 h. Lipids were extracted, and SM bands were visualized (*bottom*). *C*, the R986H mutation resulted in lower SM efflux than the WT counterpart. The K2027Q mutant did not alter SM efflux. WT and the two indicated ABCA7 mutants were expressed in 293T cells. Expression of ABCA7 proteins (*left*, *top*) and vinculin (*middle*) was detected with specific antibodies. Cells were also used to study the efflux of SM and visualized with a PhosphorImager (*left*, *bottom*) and quantified (*right*). Values are plotted as mean ± SD. ∗∗∗*p* < 0.001 compared with ABCA7-WT. *D*, representative TLC results showing diminished intracellular NBD-SM levels in cells expressing R986H mutant. In this experiment, 293T cells were transfected in triplicate with plasmids for expression of FLAG-tagged WT or R986H mutants. Cells were labeled as in panel *C* and used to visualize SM efflux. *E*–*G*, the Alzheimer’s disease–related R989H mutation in human ABCA7 decreases SM synthesis and efflux. Mouse hippocampal neuronal HT22 cells were transfected with vectors for expression of C-terminally GFP-tagged human ABCA7-WT or ABCA7-R989H proteins. *E*, cells were fixed and visualized with a fluorescence microscope. Representative single cell images indicate perinuclear and vesicular localization of the proteins. Nuclei were stained with DAPI. *F*, the expression of these two proteins after transfection was detected with a mouse anti-GFP IgG antibody. Immunoblot (IB) shows expression of these proteins. Vinculin served as a loading control. *G*, representative TLC results showing NBD-SM levels in the media (*top*) and in cells (*bottom*) transfected with the indicated expression vectors. BSA, bovine serum albumin; HDL, high-density lipoprotein; SM, sphingomyelin.

ABCA7 protein contains two each of extracellular, transmembrane, and intracellular ABC domains (10). The ABC domains (Fig. 5*A*) are important for protein function (10, 11). To address the roles of these domains in SM synthesis and efflux, we first transfected 293T cells with a control plasmid or a plasmid expressing FLAG-tagged mouse ABCA7 (Fig. 5*B*, top). Expression of ABCA7 resulted in significantly greater SM efflux than that observed in the control cells (Fig. 5*B*, bottom). Next, we expressed mouse WT ABCA7, R986H, and K2027Q missense mutations in cells (Fig. 5*C*, top). SM efflux was similar in cells expressing WT ABCA7 and the K2027Q missense mutant (Fig. 5*C*, bottom and right graph). However, SM efflux was significantly lower in cells expressing R986H than in WT cells (Fig. 5*C*, bottom and right graph). Next, we studied the effect of the R986H mutation on cellular SM levels and found that cells expressing R986H had lower levels of SM than WT cells (Fig. 5*D*). These studies showed that overexpression of ABCA7 increases SM synthesis and efflux. Expression of the R986H, but not the K2027Q, missense mutation decreased SM cellular levels and efflux, thus indicating that at least one of the ABC domain is important for cellular SM levels and efflux.

We then studied the effect of overexpression of human ABCA7 and the R989H missense mutation orthologous to mouse R986H reported in a Belgian cohort (12) that might be associated with LOAD (10, 13) on the synthesis and efflux of SM in mouse hippocampal neuronal HT22 cells. Overexpression of GFP-tagged ABCA7-R989H showed strong vesicular localization, similar to ABCA7-WT (Fig. 5*E*). In addition, similar cellular protein levels were detected with a mouse anti-GFP IgG antibody *via* immunoblotting (Fig. 5*F*). Next, we studied the effect of the human R989H mutant on intracellular and medium SM levels in cells incubated with HDL. R989H expressing cells, compared with WT ABCA7 expressing cells, had lower levels of SM in the media and in cells (Fig. 5*G*). These results indicated that the human ABCA7 missense mutant R989H is defective in SM synthesis and efflux in neuronal cells.

### ABCA7 deficiency in mice decreases SM levels in the brain

**Figure 6:**
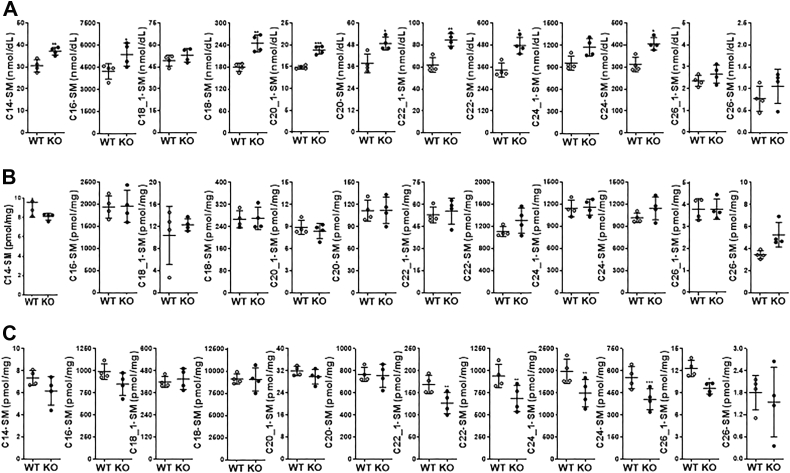
**Deletion of the *Abca7* gene in mice decreases different SM species in the brain.** Chow diet fed, 12-week-old WT, and KO male mice (n = 4) were fasted overnight, and different SM species were measured in the plasma (*A*), liver (*B*), and brain (*C*). Values are plotted as replicates (mean ± SD). ∗*p* < 0.05, ∗∗*p* < 0.01, and ∗∗∗*p* < 0.001, as compared with WT mice. SM, sphingomyelin.

KD, KO, overexpression, and mutagenesis studies in the cells described above identified ABCA7 as a protein involved in SM synthesis. Next, we extended these studies to mice and investigated the consequences of ABCA7 deficiency on the levels of different SM species in the plasma, liver, and brain. Several SM species were more abundant in the plasma of ABCA7-deficient (*Abca7*^*−/−*^, KO) mice than in WT controls (Fig. 6*A*). In contrast, we did not observe any significant change in the levels of these sphingolipids in the livers of KO mice (Fig. 6*B*). However, analysis of different species of SM revealed significantly lower levels of C22:1-, C22-, C24:1-, C24-, and C26:1-SM in the brains of KO mice than those in WT controls (Fig. 6*C*). These data suggest that ABCA7 may play a role in determining the levels of SM with longer fatty acid species in the brain.

### ABCA7-deficient mice show abnormal cognitive discrimination during the active place avoidance task

**Figure 7:**
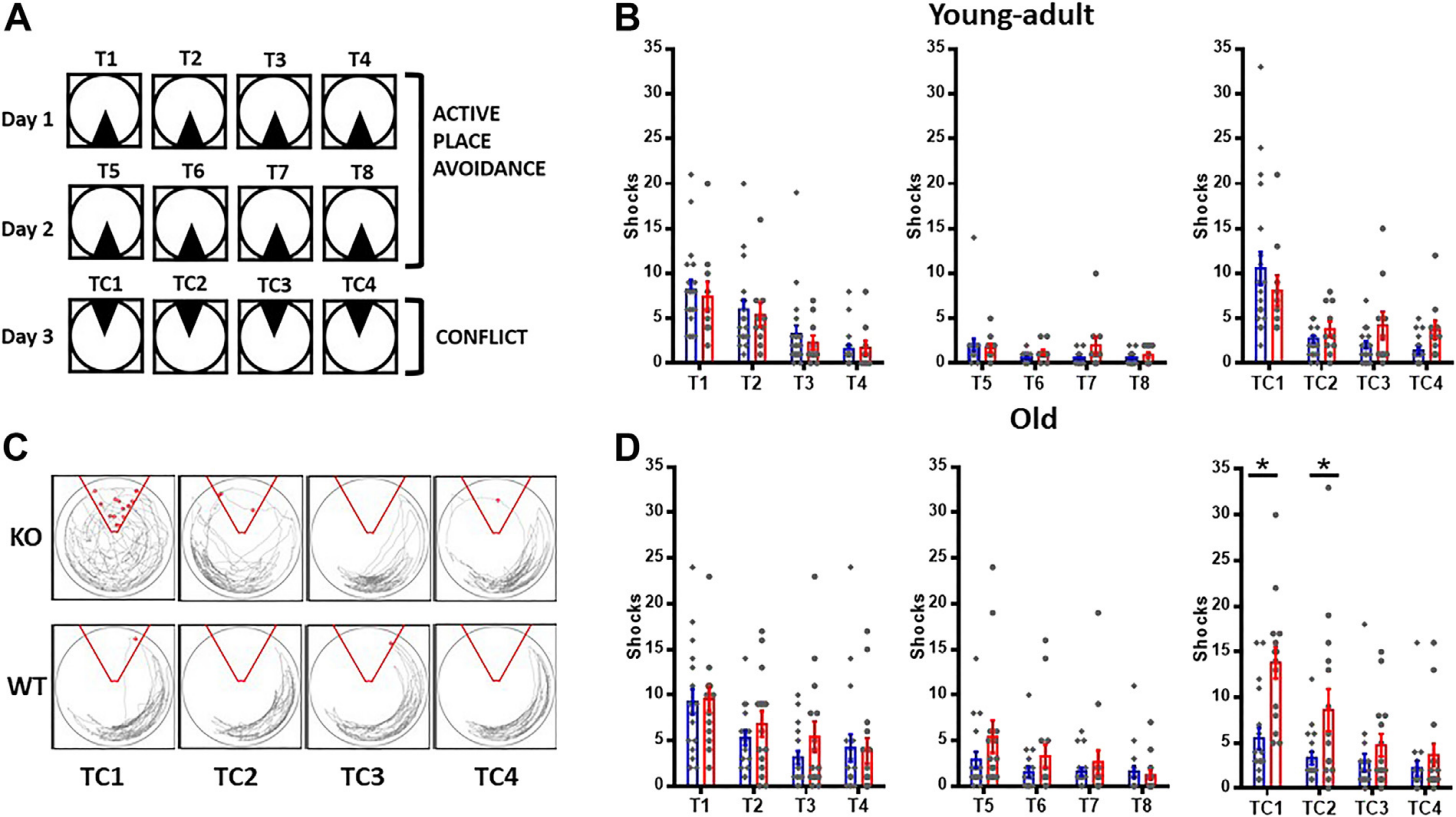
**Impaired cognitive discrimination in older male KO mice.***A*, schematic representation of active place avoidance. The *black triangle* in days 1 and 2 (*upper panels*) indicates the fixed shock zone, whereas the *open circle* indicates the rotating arena. *Lower panel* shows the configuration on day 3, in which the shock zone was rotated 180° (conflict). On day 1, all animals were habituated to the rotating platform and to the spatial fixed cues for 10 min (not shown in the figure). After 30 min, the APA task began with four 10-min trials with foot shocks in the fixed zone with intervals of 30 min between trials (T1, T2, T3, and T4). On day 2, the APA task continued with four 10-min foot shocks in the fixed zone with 30-min intervals between tasks (T5, T6, T7, and T8). On day 3, the foot shock zone was moved 180°, and conflict APA was performed with four 10-min trials with 30-min intervals (TC1, TC2, TC3, and TC4). *B*, young-adult mice (4–6 months old): quantification of shock entrances in APA in young-adult WT and KO mice, no significant differences between genotypes were found in the two-way repeated measures ANOVA (F_1,28_ = 0.1164; *p* = 0.735), (WT n = 10F/10M; KO n = 3F/7M). *C*, representative KO and WT mouse paths and shocks received during conflict trials. Representative trace of paths (*gray lines*) and number of shock entrances (*red points*) during the third day (APA conflict) in older mice. A well-defined path pattern was observed in WT, whereas a random pattern was observed throughout the trials in KO mice. KO mice entered the shock zone more often than WT mice. *D*, old mice (14–18 months old): quantification of shock entrances in APA in old WT (n = 7F/10M) and KO (KO n = 8F/7M) mice. Repeated measures ANOVA shows significant differences between genotypes (F_(1,31)_ = 4.320, *p* = 0.046) and Sidak’s post hoc test shows significant differences in both TC1 and TC2 trials (*p* ≤ 0.0001 and 0.0252 respectively). ∗*p* < 0.05. All data is presented as mean ± SEM. *Blue*, WT; *red*, KO. APA, active place avoidance.

Given the association between ABCA7 mutations and LOAD (14, 15), we used the active place avoidance (APA) task to evaluate hippocampal-dependent and hippocampal-independent behaviors (16, 17, 18) in WT and KO mice (Fig. 7*A*). Young-adult (4–6 months old) WT and KO mice quickly learned to actively avoid the shock zone, as evidenced by decreases in the number of shocks that they received in different trials (Fig. 7*B*). Older KO (14–18 months old) mice also learned to avoid the shock zone (Fig. 7*D*, T1–T8). The values of these parameters were not significantly different to those of WT mice of the same age. These studies suggested that young and old KO mice do not show any visuospatial memory disabilities as assessed by APA, in agreement with the studies of Logge *et al.* (19), who did not observe a decline in cognitive function in young-adult mice.

We then investigated cognitive discrimination by changing the location of the shock zone (APA conflict, Fig. 7*A*, TC1–TC4) (16, 17, 18). Representative recordings of mouse paths and shocks received by a KO and a WT mouse are shown in Figure 7*C*. These recordings show that older WT mice quickly learned to avoid the conflict area and displayed a regular pattern of movement within the test arena. In contrast, older KO mice received more shocks and showed a random pattern of movement in the arena. Quantification revealed a transient increase in shocks in both young-adult WT and KO mice (Fig. 7, *B* and *D*; TC1, TC4), thus indicating that both WT and KO young-adult mice learned to ignore the initial location of the shock and acquired the information on the new location of the shock zone. With continued training, both the WT and KO young-adult mice rapidly learned to discriminate between the old and new shock locations (Fig. 7*B*, TC1–TC4). However, older KO mice received significantly more shocks in the first two trials of the third day, in contrast to older WT mice (Fig. 7*D*, TC1–TC2), but eventually learned to avoid the new shock zone (Fig. 7*D*, TC3–TC4). These studies showed that KO mice required more shocks to learn the new location of the shock zone. We interpret these studies to suggest that older KO mice have cognitive discrimination deficits.

Since animals use visual cues to locate the shock zone in the APA task, we performed a visual Cliff test (20) of visual depth perception on a group of KO (n = 10) and WT mice (n = 12), >12 months old, and observed no significant differences (ANOVA safe. 8.42 ± 0.36 trials *versus* 9.33 ± 0.35; F = 1.59, *p* = 0.11). We also did not find differences in distance traveled, the number of shocks per entrance, or between males and females (Fig. S2). These studies suggest that the differences between KO and WT mice seen in APA conflict were not due to major visual abnormalities, locomotor problems, or differences in the sensitivity to the shocks. Subsequently, we attempted to address reasons for cognitive decline in older ABCA7 KO mice.

### ABCA7 deficiency impairs synaptic plasticity in the lateral entorhinal cortex but not in the hippocampal CA3-CA1 synapse

**Figure 8:**
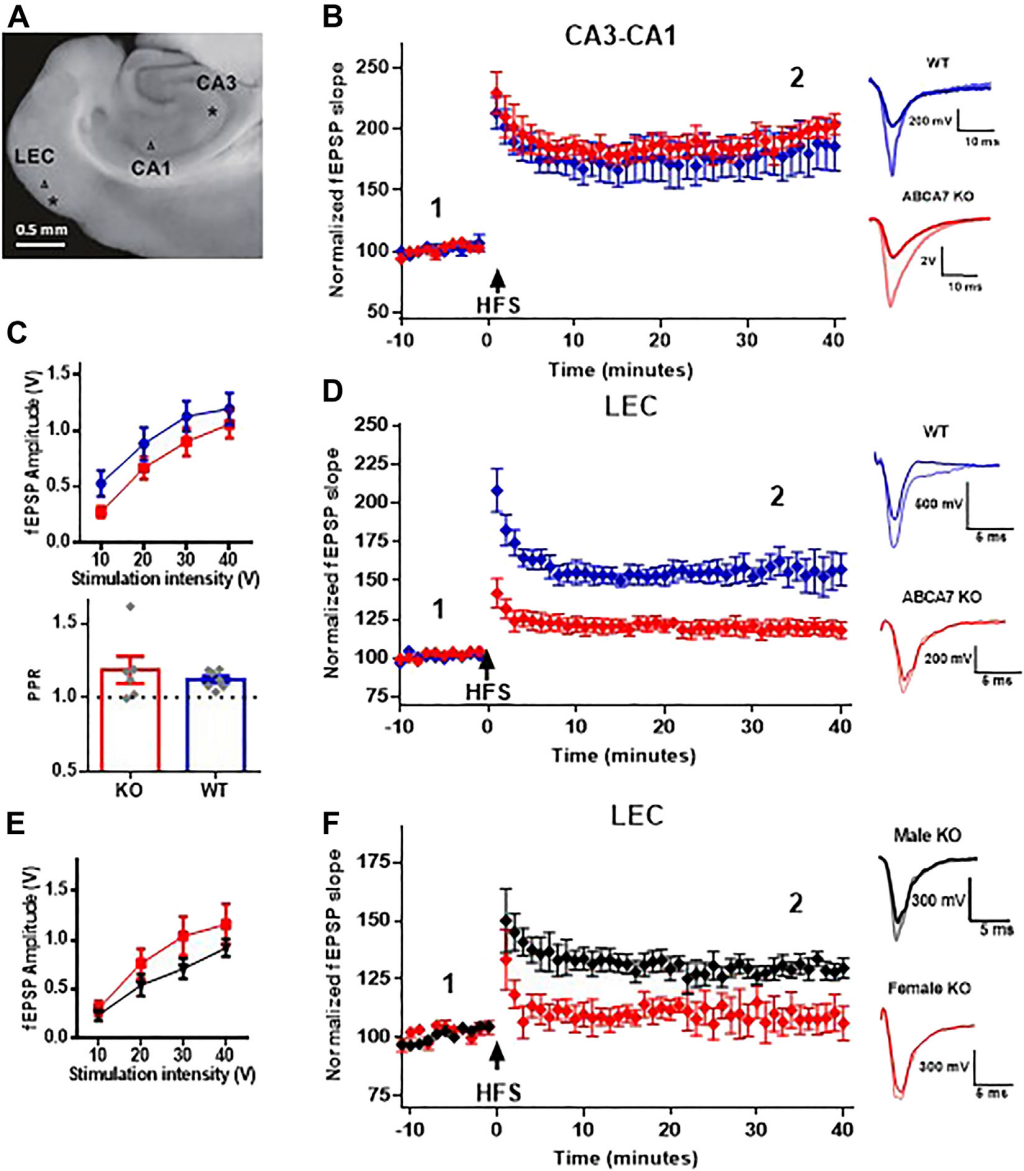
**ABCA7 deficiency impairs long-term potentiation in the lateral entorhinal cortex but not in the CA3-CA1 synapse.***A*, a representative microphotograph of the brain slices used. ∗ represents the area of the stimulating electrode (presynaptic site), and ^Δ^ represents the recording site (postsynaptic site). *B*, time course of CA3-CA1 long-term potentiation (LTP) in WT (*blue*) and KO (*red*) mice. Brain slices were obtained from 15 to 19 month old male and female mice. The Y axis shows the percentage of field EPSP (fEPSP) slope change. After a 10-min baseline, a HFS protocol (100 stimuli at 100 Hz) was used to induce LTP and responses were recorded for 40 min after HFS. Representative fEPSP traces before (1, darker trace) and 40 min after (2, lighter trace) HFS are displayed on the *right side* for WT and KO. Note that both WT and KO (n = 5 in each group) potentiated to the same extent (F_1,8_ = 0.2334 *p* = 0.6419). *C*–*F*, panels (*C*–*F*) show results in the LEC intracortical synapse. *C*, input/output (I/O) curve (*top*) and paired pulse ratio (PPR) values (*bottom*) for LEC in WT and KO mice. I/O curves are displayed as the fEPSP amplitude in response to stimulus of different intensities, repeated measures ANOVA showed no significant differences (F_1,23_ = 1.598 *p* = 0.2189; WT n = 8, KO n = 17). PPR was calculated from a pair of pulses separated by 50 ms, no significant differences were found between WT and KO mice (*p* = 0.4763; WT n = 7, KO n = 6). *D*, time course of LEC LTP in WT (*blue*) and KO (*red*) mice. After a 10-min baseline, a HFS protocol (three trains of 100 stimuli at 100 Hz were delivered with a 10-s interval between them) was used to induce LTP and responses were recorded for 40 min after HFS. Representative fEPSP traces before (1, darker trace) and 40 min after (2, lighter trace) HFS are displayed on the *right*. LTP was significantly lower in KO mice (F_1,16_ = 20.71, *p* = 0.0003; WT n = 6, KO n = 12). *E*, I/O curve for LEC in KO females (*red*) and KO males (*black*). No significant differences were found between females and males of the KO group (F_2,22_ = 1.455, *p* = 0.2549; KO females n = 10, KO males n = 7). *F*, time course of LEC LTP in KO females (*red*) and KO males (*black*) mice. After a 10-min baseline, a HFS protocol (as described in *D*) was used to induce LTP and responses were recorded for 40 min after HFS. Representative fEPSP traces before (1, darker trace) and 40 min after (2, lighter trace) HFS are displayed on the *right*. LTP was significantly lower in female KO mice (F_1,10_ = 9.765, *p* = 0.0108; KO females n = 6, KO males n = 6). All data is presented as mean ± SEM. *Blue*, WT; *red*, KO. fEPSP, field excitatory postsynaptic potentials; LEC, lateral entorhinal cortex; LTP, long-term potentiation; PPR, paired-pulse ratio.

To test whether the KO mice had abnormalities in synaptic transmission, we evaluated the long-term potentiation (LTP) form of synaptic plasticity in the hippocampal CA3-CA1 synapse (Fig. 8*A*). No significant differences were observed 40 min after LTP induction in the CA3-CA1 synapse between WT and KO slices (Fig. 8*B*). Because CA3-CA1 LTP has been proposed to be required for hippocampus-dependent memory storage (18), these results are consistent with the behavioral data (Fig. 7*B*).

Next, we evaluated the LTP in the intracortical synapse in layer II/III of the lateral entorhinal cortex (LEC). No significant differences were observed in the input-output relationships or basal paired-pulse ratio (PPR) (Fig. 8*C*). We found that both, post-tetanic potentiation (PTP) and LTP were significantly lower in KO mice (Fig. 8*D*; PTP = 141.8%; LTP = 117.9% 40 min after high frequency stimulation (HFS)) as compared with WT mice (PTP = 208.1% *p* ≤ 0.0001; LTP = 157.1% *p* ≤ 0.0001). These studies suggest that the electrophysiological differences observed in the entorhinal cortex maybe due to impaired synaptic plasticity in this region rather than differences in basal synaptic transmission.

Next, we assessed whether the LTP deficiency in ABCA7 KO mice was different between female and male mice. We did not find significant differences in input/output relationships in male and female mice (Fig. 8*E*). Interestingly, we found that female KO mice have significantly lower LTP values (F_1,10_ = 9.765, *p* = 0.0108) (Fig. 8*F*); however, Sidak’s post hoc analysis showed no significant differences 40 min after LTP induction (*p* = 0.0881). Importantly, no differences were found between females and males in the WT group (F_1,4_ = 0.04668, *p* = 0.8395) (Fig. S3). These results suggest higher susceptibility of female mice to synaptic plasticity deficits induced by the lack of ABCA7.

### SM, but not phosphatidylcholine, supplementation rescues entorhinal cortex LTP deficits in KO mice brain slices

**Figure 9:**
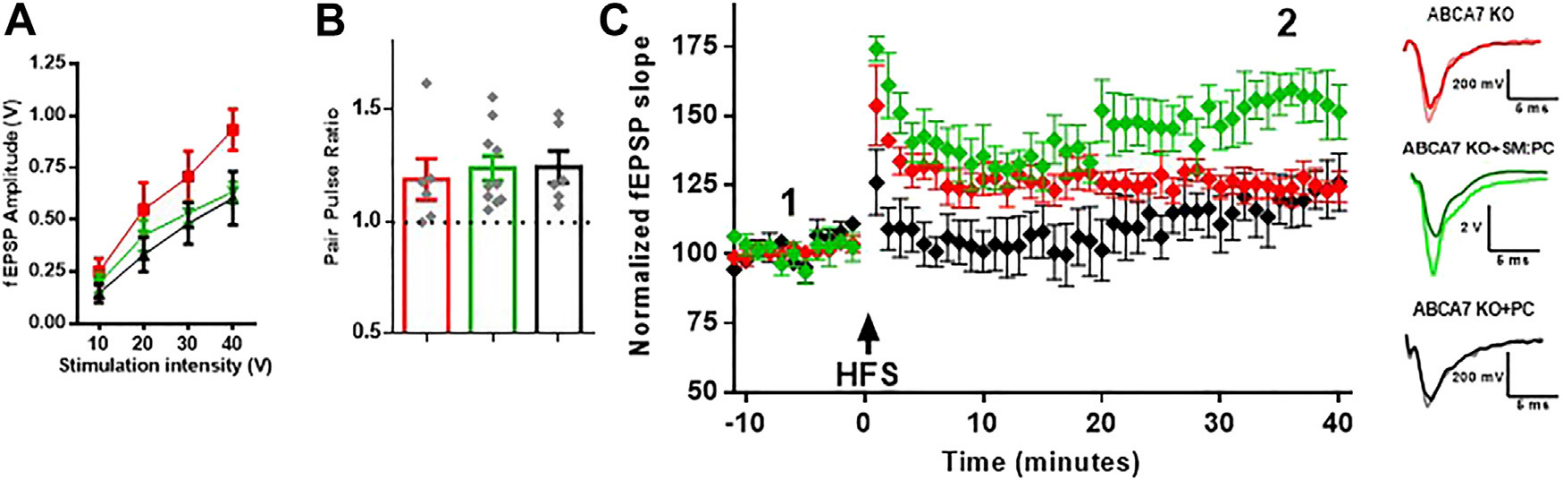
**SM supplementation rescues LEC LTP deficits in ABCA7 KO mice.***A*, input/output (I/O) curve in the LEC synapse in nontreated KO slices (*red*, KO), phosphatidylcholine (PC)-incubated KO slices (*black*, KO+PC), and sphingomyelin (SM:PC)-incubated KO slices (*green*, KO+SM:PC). I/O curve is displayed as the fEPSP amplitude in response to stimulus of different intensities, repeated measures ANOVA showed no significant differences (F_2,10_ = 1.364, *p* = 0.2993; KO n = 6, KO+PC n = 6, KO+SM:PC n = 6). *B*, paired pulse ratio (PPR) values for the LEC synapse in KO slices (*red*), KO+PC slices (*black*), and KO+SM:PC slices (*green*). PPR was calculated from a pair of pulses separated by 50 ms, no significant differences were found between the three groups (F_2,19_ = 0.1601, *p* = 0.8532; KO n = 6, KO+PC n = 6, KO+SM:PC n = 10). *C*, time course of LEC LTP in KO slices (*red*), KO+PC slices (*black*), and KO+SM:PC slices (*green*). After a 10-min baseline, a HFS protocol (three trains of 100 stimuli at 100 Hz were delivered with a 10-s interval between them) was used to induce LTP and responses were recorded for 40 min after HFS. Representative fEPSP traces before (1, darker trace) and 40 min after (2, lighter trace) HFS are displayed on the right. Repeated measures ANOVA showed significant differences between the three groups (F_2,6_ = 8.979, *p* = 0.0157; KO n = 4, KO+PC n = 4, KO+SM:PC n = 4). All data is presented as mean ± SEM. fEPSP, field excitatory postsynaptic potentials; LEC, lateral entorhinal cortex; LTP, long-term potentiation; PPR, paired-pulse ratio; SM, sphingomyelin.

Because we observed decreased levels of certain SM species in KO brains (Fig. 6*C*), and SM has previously been proposed to be involved in postsynaptic receptors trafficking, which is necessary for LTP (21, 22, 23), we asked whether the abnormalities in LEC long-term plasticity might be reversed by SM supplementation. Brain slices from KO mice were subjected to three treatments; controls, PC liposomes, and PC: SM liposomes. No significant differences were found between the three groups for the values of the I/O curve values (Fig. 9*A*) or in PPR (Fig. 9*B*). As shown in Figure 9*C*, LTP was not fully induced by HFS at the LEC region in slices obtained from KO mice (124.3% 40 min after HFS *versus* 157.7% in WT mice *p* = 0.0384). The supplementation of PC reduced the early phase of the LTP as it significantly decreased the magnitude of PTP (125.9% *versus* 153.7% in nontreated KO slices, *p* = 0.0013) and potentiation values up to 14 min after induction (107% *versus* 126.1% in nontreated KO slices, *p* = 0.0411). But potentiation was similar 40 min after induction (126% *versus* 124.3% in nontreated KO slices). However, slices from the same animals preincubated in 20 μM liposomes containing SM and PC (SM:PC) for 30 min before the recordings expressed LTP with values closer to WT levels (151.4% 40 min after HFS *versus* 157.7% in WT mice *p* = 0.999) and significantly higher PTP values (174.2% *versus* 153.7% in nontreated KO slices *p* = 0.0253). Together, these data suggest that disrupted LEC synaptic plasticity in KO brain slices can be rescued by exogenous addition of SM.

## Discussion

We previously showed that MTP and ABCA1 play important roles in determining plasma concentrations of SM (4, 5). Mechanistic studies revealed that neither of these proteins affects SM synthesis. We showed that MTP transfers SM *in vitro* between vesicles and suggested that it might also do so in cells, thus facilitating secretion of SM with lipoproteins. ABCA1 is involved in the efflux of phospholipids and cholesterol to HDL, and its deficiency in humans and mice is associated with low levels of plasma SM (5). Although chronic ABCA1 deficiency significantly decreases plasma SM levels in humans and mice, acute knockdown or inhibition has no effect on SM efflux from human hepatoma cells (5). Therefore, we concluded that ABCA1 does not play a role in SM efflux to HDL and attempted to identify the SM transporter(s). Using a siRNA library, we identified ABCA7 as a candidate protein in SM efflux. However, subsequent studies showed that ABCA7 plays a role in the biosynthesis of SM and decreased efflux to HDL might be secondary to lower cellular SM levels. These studies show that different approaches are required to identify transporters that affect only efflux without affecting SM synthesis. In one approach, cells can be enriched with labeled SM as in Figure 4 to identify efflux transporters independently of their effects on SM synthesis. Our SM supplementation studies indicated that cells take up SM from extracellular environment. Thus, this method could also be used to identify cellular acceptors that mediate this uptake.

SM, along with cholesterol, forms unique membrane subdomains termed “lipid rafts”. Lipid rafts serve as organizational structures in signal transduction, as cell signaling is disrupted when lipid rafts are disassembled with lipid scavenging molecules (24). Decreased SM levels due to ABCA7 deficiency may alter lipid rafts composition in the plasma membrane, which may affect synaptic transmission and plasticity, given that neurotransmitter receptors function and trafficking is associated with lipid rafts composition (25). Here, we report specific synaptic plasticity dysfunction in LEC, but not in the hippocampal CA3-CA1, in KO mice. We also report that this synaptic plasticity dysfunction in the LEC is more pronounced in females than in males (Fig. 8*F*).

Evidence from the literature indicates that impaired sphingolipid metabolism contributes to AD (26, 27, 28). Metabolomics studies in human cohorts have reported abnormal plasma SM levels in patients with AD (29, 30, 31). Liu *et al.* (31) reported decreases in several SM species in AD patients. Chai *et al.* (32) found the strongest association of plasma d18:1/24:1 dihexosylceramide levels with *ABCA7* gene. Han *et al.* (29) have shown lower levels of SM with 22 and 24 carbon atoms in the brains of patients with AD than in controls. Sakae *et al.* (33) showed significantly reduced levels of two SM species in the forebrain of KO mice. Consistent with our studies, all these studies point to a defect in SM metabolism in AD and association with loss of function ABCA7. Decreases in SM levels in human brains have been attributed mainly to increased sphingomyelinase enzyme activity (34). Our studies showed that ABCA7 deficiency does not affect SM uptake and hydrolysis. Instead, our studies indicate a defect in SM biosynthesis.

Rodents learn the APA task rapidly, and this learning is affected by alterations in several brain structures, such as the hippocampus and entorhinal cortex (17). Mice learn the shock zone location by attending to relevant distal visual cues in the room while ignoring irrelevant proximal olfactory cues deposited on the arena (35). Our studies showed normal visuospatial memory but abnormal cognitive discrimination in older KO mice. Hence, our findings suggest that ABCA7 deficiency disrupts electrophysiological and cognitive functions that are reminiscent of LOAD.

Our studies showed that ABCA7 deficiency has a brain-specific phenotype, and SM levels are low in the brains of KO mice. On the basis of ENCODE data, it has been suggested that ABCA7 expression is regulated differently in the brain than in other tissues (36). The reasons for the predominant effects of ABCA7 deficiency in the brain might be associated with the unique need for SM in synaptic transmission (37). SM synthesis is likely to be higher in the brain than in other tissues, and this process is augmented by ABCA7. Mechanistically, ABCA7 may act as a cofactor for enzymes involved in SM biosynthesis in the brain. Alternatively, it might be involved in the transport of SM from the Golgi to the plasma membrane, thereby avoiding product inhibition. In the absence of ABCA7, SM levels may build up in the Golgi and inhibit SM production. Additionally, ABCA7 may modulate the ceramide synthase 2 enzyme, which is involved in the synthesis of ceramides with long chain fatty acids. Ceramide synthase 2 deficiency affects myelination and cognitive function (38). More studies are needed to unravel how ABCA7 modulates and participates in brain SM metabolism, and why its deficiency decreases specific species of SM.

In the liver, in contrast to the brain, ABCA7 deficiency does not affect SM levels. Hepatic SM synthesis is likely to occur at lower levels and may not require an enhancer. SM deficiency, specifically with long chain fatty acids, in brain cells maybe secondary to high expression of ABCA7 in this tissue. Unexpectedly, the plasma of KO mice had higher levels of SM; we are currently unsure of the reason for this elevation.

A limitation of our studies is that we assumed that efflux to BSA is basal and treated it as a control. This efflux might conceivably be physiologically significant—a possibility to be addressed in the future. Another caveat is that we focused on efflux to HDL. Other acceptors for SM probably exist. In our screening studies, we used human hepatoma cells. Given the current knowledge regarding the importance of SM in brain cells, similar screening in neuronal cells by using cerebrospinal fluid HDL might identify additional transporters.

Supplementation with exogenous SM rescued LTP abnormalities in the LEC, thus suggesting that cellular SM levels, either in the rafts or at other sites in the plasma membrane, can be altered by the availability of SM in the environment and consequently affect electrophysiological properties of the brain. This finding supports the possibility that therapeutic and/or dietary interventions that enhance ABCA7 activity and/or SM levels in the entorhinal cortex might prevent defects in synaptic transmission and delay LOAD. A caveat of these studies is that we do not know where SM was acting. Most likely, it was delivered everywhere including neuronal soma, dendrites, axons, glia etc. More selective studies using isolated cells or synaptoneurosomes may yield information about the cells involved in these processes. We are uncertain as to why CA3-CA1 transmission was not impacted by ABCA7 deficiency. It is possible that this region may have low ABCA7 expression, lower dependence on SM, or both. Since we did not see any effect on CA3-CA1 transmission in ABAC7 KO slices, we did not do SM supplementation studies.

In summary, we identified ABCA7 as a player in the biosynthesis and efflux of SM. We observed that KO mice have diminished levels of SM in the brain, cognitive deficits, and that ABCA7 deficiency affects synaptic plasticity, whereas this effect is rescued by SM supplementation. These studies provide a possible mechanistic explanation for the association of LOAD with ABCA7 loss-of-function mutations and suggest that increasing brain SM may ameliorate the risk of LOAD.

## Experimental procedures

### Materials

C6-NBD Cer (6-((N-(7-nitrobenz-2-oxa-1,3-diazol-4-yl)amino)caproxyl)sphingosine, catalog #6224) was purchased from Setareh Biotech. N-palmitoyl-D-erythro-sphingosine [palmitoyl-1-[^14^C]-Cer (C16-[^14^C]-Cer, 50–60 mCi/mmol, 1.85–2.22 GBq/mmol, catalog #ARC 0831–50) was from American Radiolabeled Chemicals, Inc. All other chemicals and solvents were obtained from Fisher Scientific or VWR International.

### Mice

The KO mice (39) were backcrossed to a C57BL6/J background for approximately 15 generations. Mice were bred and housed in independently ventilated cages at Animal Bioresources (SUNY Downstate Medical Center), in compliance with all requirements of the SUNY/NIH IACUC. Young-adult (4–6 months old) and older (14–18 months) mice were used in these studies. Mice were pair-housed under a 12:12 h light: dark schedule [light phase: white light (illumination: 124 lux); dark phase: red light (illumination: <2 lux)]. Food and water were available *ad libitum*. Animals were anesthetized using isoflurane. All animal procedures were approved by the Institutional Animal Care and Use Committee of SUNY Downstate Medical Center (protocol number 15-10475).

### Quantification of lipids

For targeted lipidomics, plasma (100 μl), liver (2 mg), and brain (2 mg) samples from WT and KO (n = 4) mice were used for the quantification of different species of SM with HPLC-MS/MS (4, 5).

### Site-directed mutagenesis of the ABCA7 plasmid

The plasmid pABCA7-GFP for expression of human ABCA7 with an EGFP tag at the C-terminus was described previously (40). R989H mutation was introduced in pABCA7-GFP with a Q5 site-directed mutagens kit E0552S, (#New England Biolab) with the forward primer 5′-CGAGAAGGTC**A**CACGCTGATC-3′, the reverse primer 5′-GTATTTGAGCAGCAGCTC-3′ (Genewiz), and an annealing temperature (T_a_) of 62 °C, according to the manufacturer’s protocol. The resulting plasmid was amplified in *Escherichia coli* strain DH10b and purified with an Endo-free Plasmid Midi Kit (#D6915-03, Omega Bio-Tek). The mutated single nucleotide from G to A (underlined above) corresponding to human SNP rs115536223 was confirmed with Sanger sequencing. The plasmid for expression of mouse ABCA7 was as previously described (41). R986 (Fp 5′-AGAGAAGGTC**AT**ACACTGATTCTC-3′, Rp, 5′-GTACTTAAGTAGCAATTCC-3′, T_a_ 56 °C) and K2027 (Fp 5′-TCAGCATCTC**G**AAGGCAGGTTC-3′, Rp, 5′-GAGCTTCCCAGACAGCGG-3′, T_a_ 66 °C) were also generated as described above for human ABCA7.

### Identification of proteins involved in sphingomyelin efflux and synthesis

Human hepatoma Huh7 cells were grown in Dulbecco’s modified Eagle’s medium (DMEM) supplemented with 10% fetal bovine serum (FBS), L-glutamine, and antibiotics. Cells were reverse transfected with 50 nM *siCTRL* or siRNA against various membrane transporters (obtained from Dharmacon, GE Life Sciences) with RNAiMAX (Thermo Fisher, #13778150) according to the manufacturer’s instructions for 72 h siRNAs.

Mouse hippocampal neuronal HT22 cells were transfected with vectors for expression of C-terminal GFP-tagged ABCA7-WT and ABCA7-R989H proteins. Transfection was performed with Turbofect reagent (#R0532, Fisher Scientific) according to the manufacturer’s instructions. Cells were fixed and visualized under fluorescence microscopy to determine the localization of the proteins. Expression of proteins after transfection was detected with mouse anti-GFP IgG antibody (#632381, Clontech) (42). The endogenous level of vinculin was detected as an internal control using a mouse monoclonal antibody (#V9131, Sigma).

### Sphingolipid efflux and synthesis

For efflux, Huh7 or HT22 cells were incubated with 2 μM C6-NBD Cer in DMEM with 10% FBS at 37 °C for 3 h and washed three times with DMEM plus 0.1% BSA. Efflux was initiated by the addition of fresh DMEM containing 40 μg/ml of either BSA or human serum HDL (#MBS173147, MyBioSource). In some experiments, Huh7 cells were also incubated with 0.2 μM [^14^C]-Cer, instead of C6-NBD Cer, to study sphingolipid synthesis and efflux. Culture media were harvested after 1 h or 6 h of incubation and centrifuged (2500 rpm, 600*g*, 15 min, 4 °C, Heraeus Fresco 21 Centrifuge, rotor 75003424, Thermo Fisher Scientific) to pellet detached cells. Lipids in the media and cells were extracted (43), dried, and resuspended in 100 μl of isopropanol for the separation of sphingolipids on TLC silica plates (#44931, Analtech, Inc) with a CHCl_3_:CH_3_OH:C_6_H_5_CH_3_:NH_4_OH:H_2_O (40:40:20:0.4:1.6, ratio by volume) solvent system (4, 5). The TLC plates were visualized with a PhosphorImager (GE Healthcare), and bands corresponding to Cer, GluCer, and SM were quantified in ImageJ software.

### Uptake and metabolism of sphingomyelin in primary hepatocytes

Primary hepatocytes from WT and KO mice were isolated as described previously (44). Primary hepatocytes were cultured overnight at 37 °C in HepatoZYME-SFM (Gibco Cat. No. 17705-021) containing 10% FBS. After overnight culture, the medium was replaced with fresh HepatoZYME-SFM (10% FBS) containing 2 μM C6-NBD-SM (#810218C, Avanti Polar Lipids, Inc). Culture medium was removed at different time points (0, 15, 30, and 45 min) and cells were washed with PBS. To determine uptake of SM and generation of ceramide, 500 μl of isopropanol was added to each well and incubated overnight at 4 °C. Next day, isopropanol was collected and dried for lipid measurements. To each well, 500 μl of 0.1 N NaOH was added to determine protein concentration. After protein normalization, dried lipids were resuspended in required volumes of isopropanol. One-fifth amount of samples in isopropanol were used for total cellular NBD fluorescence readings and rest of the lipid samples were separated on thin layer silica plates (Merck #1.05554, TLC Silica gel 60 F_254_) using a CHCl_3_/CH_3_OH/C_6_H_5_CH_3_/NH_4_OH/H_2_O (40:40:20:0.4:1.6, ratios by volume) solvent system. The TLC plates were visualized in a Typhoon FLA 9500 laser scanner (GE Healthcare), and the NBD-lipid molecules were quantified using Image J software. The NBD fluorescence readings were measured as described previously (45).

For SM metabolism studies, WT and KO primary hepatocytes were incubated with 2 μM C6-NBD-SM in HepatoZYME-SFM with 10% FBS at 37 °C for 4 h, medium was removed, cells were washed with PBS, and replaced with fresh HepatoZYME-SFM with 10% FBS. Culture medium was collected at different time points (0, 15, 30, and 45 min) and cells were washed with PBS. To each well, 500 μl of isopropanol was added and culture plate was incubated overnight at 4 ° C. Lipid extraction, TLC analyses, and total cellular NBD fluorescence readings were performed as mentioned above.

### Measurement of Abca7 and Cert1 transcript levels

For Abca7 and Cert1 quantification by quantitative RT-PCR, cDNA was synthesized from isolated RNA with a high-capacity cDNA reverse transcription kit (Thermo Fisher Scientific, #4368813).

The mouse Abca7, Cert1, and 18S-specific primers were purchased from Integrated DNA Technologies (Abca7 primers, 5′-CGCACATTGGCAGAGATTCG-3′ and 5′-ATCTTGAGGCTGTTCCGAGC-3′; Cert1 primers, 5′-CCTCGCTGAGCGTGTAACTA-3′ and 5′-TTTCACTCCAAGGAGCAAGCA-3′; 18S primers, 5′-GATCCGAGGGCCTCACTAAAC-3′ and 5′-AGTCCCTGCCCTTTGTACACA-3′). For quantitative RT-PCR, PowerTrack SYBR Green Master Mix (Thermo Fisher Scientific, #A46109) was used. For Abca7 and Cert1 quantification, the Ct method with normalization to 18S was used and data are presented as fold change as described previously (46).

### APA response in KO mice

KO and WT mice underwent 3 days of APA testing to assess the ability of the mice to segregate sensory information and learn the location of a stationary shock zone on a rotating arena (16, 17). Behavioral assessments were conducted in a rectangular room (4 m × 3 m) containing an apparatus consisting of a 40-cm diameter circular arena that rotated at 1 rpm. The room had visual landmarks on the walls. A computer tracked the position of the mouse with a computer-controlled infrared FireWire camera located 1.2 m above the arena. The signal from the camera was analyzed with a spot-tracker (BioSignal Group). The movement of the animal relative to the arena was calculated every 33 milliseconds (ms) from the positions of the mouse and a light emitting diode that marked the outer part of the arena. During APA, the computer defined a 60° segment of the arena as the shock zone. Entry into the shock zone for 500 ms triggered a shock of constant current (500 ms, 60 Hz, 0.2 mA) delivered through the grid floor. Additional shocks were administered every 1.5 s until the mouse left the shock zone. Track analysis software (Bio-Signal Group Corp) was used to objectively analyze the movement of the mouse and the number of entries into the shock zone. Mice were habituated to handling and the training environment. Mice first received a 10-min habituation/open field session with the shock zone turned off. Total distance traveled was assessed. Mice had 2 days of 4 × 10 min sessions of APA with an active shock zone. The total distance traveled, speed, linearity, shocks per entrance, number of shock zone entrances, and time to first entrance were assessed. On day 3, the shock zone was rotated 180° (conflict), and similar 4 × 10 min sessions were run with evaluation of the same parameters. Two-way repeated measures ANOVA and post hoc SIdak’s test were used to analyze APA data, and normal distribution of the data was confirmed with a D'Agostino & Pearson omnibus normality test.

### PC and PC:SM vesicle preparation

For PC and PC:SM vesicle preparation, egg PC (L-α-phosphatidylcholine 95%; #131601C) and egg SM (#860061C) were purchased from Avanti Polar Lipids. For preparation of 20 mM PC vesicles, 3.08 ml of PC (25 mg/ml stock) was added to a 15 ml glass tube, and chloroform was completely evaporated under the flow of nitrogen. Similarly for 20 mM PC:SM vesicles, 3.08 ml of PC and 2.84 ml of SM (25 mg/ml stock) were mixed, and chloroform was completely evaporated under the flow of nitrogen. To each dried sample, 5 ml molecular grade PBS was added, and glass tubes were vortexed to mix and dissolve the lipids properly. Then each sample was sonicated for 3 h with a Fisher Scientific 550 Sonic Dismembrator on ice to avoid heating of the sample. Samples were manually monitored intermittently for heat and clarity. After sonication, the cloudy, whitish samples become clear. To remove invisible particles, samples were transferred to 1.5 ml tube and centrifuged (5424 R centrifuge, FA-45-24-11 rotor, 20,000*g*, 20 min, 4 °C). After centrifugation, the clear samples were collected in 15 ml tubes and stored at 4 °C.

### Slice electrophysiology

All electrophysiology experiments were performed 1 month after behavioral tests. Ventral hippocampal horizontal slices (400 μm, consisting of ventral hippocampus; subiculum complex; and entorhinal, perirhinal, and temporal cortices) (47) were obtained as previously reported (48). Briefly, mice were anesthetized with isoflurane and decapitated; brains were removed and sectioned through the ventral hippocampus into 400 μm-thick slices in ice-cold cutting solution (in mM: K-gluconate 130, KCl 15, Hepes acid 20, glucose 25, kynurenic acid 50 μM, and EGTA-K 50 μM. pH 7.4) bubbled with O_2_ and CO_2_. Slices were then incubated in O_2_ and CO_2_ bubbled artificial cerebrospinal fluid (aCSF; in mM: 130 NaCl, 2.5 KCl, 2 CaCl_2_, 1 MgCl_2_, 1.3 NaH2PO4, 26 NaHCO_3_ and 10 glucose) at 34 °C for 30 min and allowed to recover at room temperature for at least 30 min.

Evoked field excitatory postsynaptic potentials (fEPSP) were recorded in the CA1 stratum radiatum with glass electrodes filled with 150 mM NaCl (2–3 MΩ resistance) and were elicited by stimulation of the Schaeffer collateral fibers with a tungsten bipolar electrode. Recordings were performed with an Axoclamp 2B amplifier (Molecular Devices) and filter (0.1 Hz–10 KHz with −6 dB/octave). Voltage signals were digitized and stored on a computer with a Digidata 1200 A digitizer (Molecular Devices) for offline analysis. Data were analyzed with Clampfit (Molecular Devices). Input-output relationship curves were obtained, and a stimulus evoking ∼40% of the maximum response was selected for the LTP experiments. A baseline response was obtained (15 min with an inter-stimulus interval of 20 s), and a high-frequency stimulation (one train of 100 stimuli at 100 Hz) was used to induce synaptic LTP. Responses were recorded for 50 min after induction. The slope (10–90%) of the fEPSP was measured and expressed as a percentage of baseline (48). The results are expressed as mean ± SEM.

For the LEC-LEC synapsis, recordings were performed by stimulating the LEC II with a bipolar electrode and recording in LEC III/II with a glass electrode filled with 150 mM NaCl. An input-output relationship curve was obtained, and a stimulus evoking ∼40% of the maximum fEPSP response was selected for PPR and LTP experiments. PPR assessment was performed by delivering two pulses separated by 50 ms, maximum amplitude was measured for both fEPSP, and the PPR was calculated by dividing the amplitude of the second fEPSP by the amplitude of the first fEPSP. For the LTP induction, one train of high frequency stimulation (100 pulses at 100 Hz) was applied every 10 s three times (49), and responses were recorded for 50 min after induction, measured as fEPSP slope (10–90%), and expressed as a percentage of baseline, as previously described (50). To ensure that the elicited fEPSP was indeed glutamatergic, at the end of each recording, NMDA- and AMPA-mediated currents were blocked with 30 μM (2*R*)-amino-5-phosphonovaleric acid and 10 μM 2,3-dihydroxy-6-nitro-7-sulfamoyl-benzo[f]quinoxaline-2,3-dione.

For PC and SM:PC vesicle supplementation, brain slices were incubated in this solution for 30 min and vesicles were added at a 20 mM concentration and immediately moved to the recording chamber with regular aCSF perfusion for electrophysiology experiments. Whenever possible, PC, SM:PC, and nontreated KO reported recordings were performed in slices from the same mouse. Data were analyzed with repeated measures ANOVA and Sidak’s post hoc test for multiple comparisons between two groups and Tukey’s post hoc test for multiple comparisons between three groups. PPR data were analyzed with Student’s *t* test between KO and WT and with one way ANOVA for supplementation experiments.

### Statistics

Behavioral and electrophysiological data were analyzed with repeated measures ANOVA or one way ANOVA and Sidak’s or Tukey’s post hoc analysis. Biochemical data between WT and KO mouse models were analyzed with Student’s *t* test. Comparisons among different siRNA treatments were evaluated with Student’s *t* test. All statistical analysis was performed in GraphPad Prism.

## Data availability

All the data is in the article.

## Supporting information

This article contains supporting information.

## Conflicts of interest

The authors have no conflicts to declare with the contents of this article.
