# Supplementary material for: ATP-binding cassette protein ABCA7 deficiency impairs sphingomyelin synthesis, cognitive discrimination, and synaptic plasticity in the entorhinal cortex
Source: J Biol Chem. 2022 Aug 23;298(10):102411. doi: 10.1016/j.jbc.2022.102411 (PMC9513280; doi:10.1016/j.jbc.2022.102411)
Supplement: Supplemental Figures S1–S3 [file mmc1.docx]

**Supplementary Figures**

Supplementary Figure 1

**Supplementary Figure 1.** Expression of ABCA7 and CERT1 mRNA levels in ABCA7 deficient mice. Livers (n=3) were collected from chow fed male WT and KO mice and mRNA levels of ABCA7 and CERT1 were quantified. Average ± SD, n=3, student *t*-test, ^**^p<0.01.

Supplementary Figure 2

**Supplementary Figure 2.**

A) Distance traveled in the APA task, showing no significant differences between genotypes (F (1, 32) = 0.3694 p=0.54; WT n=19, KO n=15).

B) Number of shocks per entrance calculated by dividing the number of shocks over the number of entrances to the shock zone. No significant differences were found between genotypes (F (1, 31) = 0.2058 p=0.65; WT n=18, KO n=15).

C) Number of shocks in KO males vs WT males. No significant differences were found between genotypes with two-way repeated measures ANOVA (F (1, 15) = 2.519 p=0.13), however significant differences were found in the post hoc analysis in conflict trials (TC1 p=0.0002; TC2 p=0.009) (WT males n=11, KO males n=7).

D) Number of shocks in KO females vs WT females. No significant differences were found between genotypes with two-way repeated measures ANOVA (F (1, 13) = 2.206 p=0.16), however significant differences were found in the post hoc analysis in conflict trials (TC1 p=0.028) (WT females n=7, KO females n=8).

Supplementary Figure 3

**Supplementary Figure 3.** Time course of LEC LTP in WT females (blue) and WT males (black) mice. After a 10-minute baseline, a HFS protocol (3 trains of 100 stimuli at 100Hz were delivered with a 10-second interval between them) was used to induce LTP and responses were recorded for 40 minutes after HFS. no differences were found between WT females and WT males (F _1, 4_ = 0.04668, p=0.8395). (WT female n=3; WT male n=3).
